# Superior Dislocation of the Patella in a Young Patient without Osteophytes: A Case Report with Discussion about Differential Diagnosis

**DOI:** 10.1155/2019/7314698

**Published:** 2019-03-27

**Authors:** Wander Edney de Brito, Gustavo Constantino Campos, João Batista de Miranda, Alessandro Rozim Zorzi

**Affiliations:** ^1^São Leopoldo Mandic School of Medicine, Campinas, Brazil; ^2^Hospital Municipal Mário Gatti, Campinas, Brazil; ^3^State University of Campinas (UNICAMP), Campinas, Brazil; ^4^Hospital Israelita Albert Einstein, Sao Paulo, Brazil

## Abstract

We report a case of superior dislocation of the patella in a young woman without degenerative changes. We retrospectively analyzed the clinical and imaging data obtained from the patient. This article describes a rare case of patellar dislocation following a bicycle fall in a 19-year-old woman without any history of patellofemoral complaints. Our literature search yielded 28 case reports; however, most reports describe older individuals with osteoarthritis. Only two reports have previously described this lesion in young patients without osteophytes, but some features, like an increase of the patella tilt, may raise doubts about whether it would be better to classify them as a vertical dislocation of the patella, another quite rare lesion, or just as a variant of a superior dislocation.

## 1. Introduction

Superior dislocation of the patella (SDP) is a rare condition that is often associated with degenerative changes and the presence of osteophytes, with peak incidence observed in the sixth decade of life [1]. The distal articular edge of the patella engages in the proximal articular edge of the trochlea, locking the knee in full extension.

Although rare, this condition requires urgent treatment. Patients with SDP often present to the Emergency Department (ED) with severe pain and restriction of movement. It is important to not misdiagnose SDP as an acute rupture of the patellar tendon based on radiographic findings. Careful history taking and clinical examination are important to differentiate between these conditions [2]. SDP requires immediate closed reduction, whereas patellar tendon rupture requires surgical treatment in a nonemergency setting.

This case report describes one of the youngest known patients diagnosed with SDP.

## 2. Case Presentation

Ethical approval and informed consent to report this case and its figures were obtained from the Ethics Committee of the State University of Campinas (approval number 2.878.038/ID 95776318.8.0000.5404).

**Figure 1:**
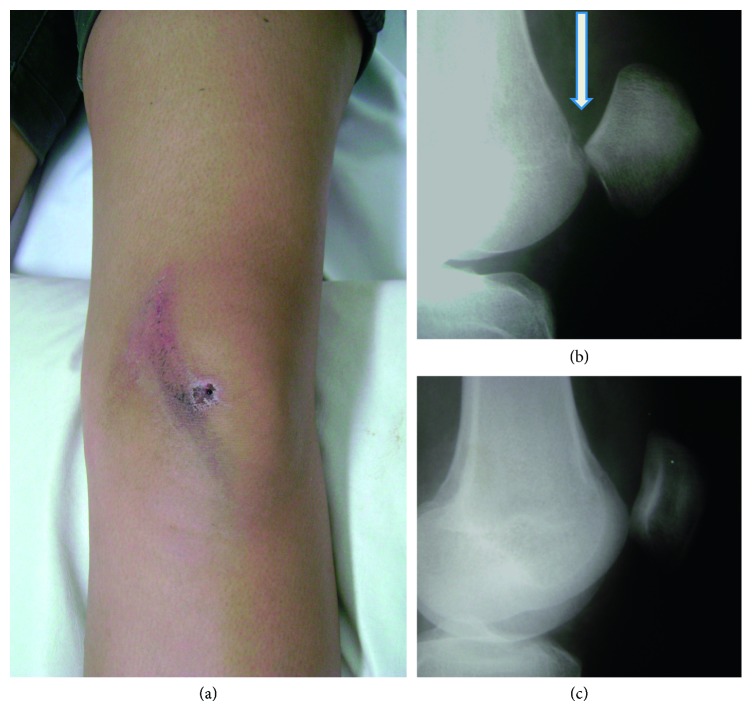
Image shows the patient's left knee upon arrival at the ED, after a fall from a bicycle (a). It is possible to notice that there is no bulging in the anterior region of the knee found in the VDP. SDP is suspected based on the attachment of the distal end of the patella to the proximal end of the trochlea (arrow). The high riding patella needs to be distinguished from a patellar tendon rupture (b). Radiograph of the left knee obtained after the procedure shows successful reduction (c). ED: emergency department; SDP: superior dislocation of the patella.

A 19-year-old woman fell from a bicycle and hit her knee against a street guide. She presented with severe pain and an anteromedial skin bruise on the left knee and her knee locked in full extension (Figure 1(a)).

Radiographs showed superior displacement of the patella with its inferior articular surface engaging the proximal articular surface of the trochlea (Figure 1(b)).

We administered 2% lidocaine hydrochloride intra-articularly to treat her pain. Superior-inferior and lateral-lateral patellar manipulations were unsuccessful, and we subsequently grasped and anteriorized the patella in relation to the femur, which led to immediate reduction. A plain control radiograph was obtained following this procedure and showed complete reduction of the patellofemoral joint (Figure 1(c)).

To ensure patient comfort, the knee was immobilized for a week in an inguino malleolar orthosis at 20 degrees of flexion. Upon her return 10 days later, the patient was completely pain free and asymptomatic without any movement limitations.

## 3. Discussion

Watson-Jones first described SDP in 1946 [3]. All reports since then have described SDP in older patients presenting with trochlear and patellar osteophytes causing engagement of the osteophytes at the lower pole of the patella and at the proximal trochlea [4]. Other authors have reported similar cases in old patients with knee osteoarthritis, always with locking osteophytes.

In 2007, Saleemi et al. [5] first reported SDP in a young patient with no radiographic evidence of osteoarthritis and no osteophytes. In 2016, Kataoka et al. [6] described the second case in a 19-year-old woman who developed SDP secondary to direct knee trauma. Our case is the third report describing SDP in young patient without osteophyte.

However, it is noteworthy that, in our case and in the two previously cited [5, 6], the patella presents an increased tilt in the lateral radiography, which does not occur in cases with presence of osteophyte. This can cause confusion with another very rare lesion, the vertical dislocation of the patella (VDP) [7, 8]. Would these cases be better classified as VDP? In this way, there would be no cases of SDP without osteophytes. According to van Egmond et al. [4], there is a pathognomonic signal of the SDP: the proximal part of the patella is tilted away from the femur. This is caused by the pull of the patella tendon and the simultaneous relaxation of the quadriceps tendon. Kataoka et al. [6] performed a CT scanning showing clearly that, despite the increased patella tilt and the absence of a higher patella height, there is the attachment of the articular surface of the distal patella in the proximal trochlea, which is a characteristic of the SDP. Another interesting fact is that, in the VDP images, it is possible to notice an exuberant protrusion in the anterior region of the knee [8, 9]. Based on these three arguments, we therefore chose to classify our case as SDP, but we agree that there are differences in relation to the classical SDP described in patients with osteophytes.

Clinicians must consider SDP among the differential diagnosis in patients presenting to the ED with direct trauma to the knee associated with anterior knee pain. Initial radiographs may misdiagnose SDP as a ruptured patellar tendon [10, 11]. Careful examination is important to identify locking of the knee in full extension. Ultrasonography is a useful diagnostic aid in doubtful cases [11, 12].

Several maneuvers have been described to perform closed reduction of the patella. Usually, intra-articular administration of local anesthetics provides sufficient analgesia; however, sedation or regional blocks may be indicated. The knee should be positioned in hyperextension with the hip flexed to relax the rectus femoris muscle. The patella is secured between the operator's two digits. Gentle lateralization and medialization movements are performed until successful reduction occurs. If this maneuver does not yield results, forceful anteriorization of the patella can be attempted. However, open reduction is required in patients not responding to these aforementioned maneuvers [13, 14].

## 4. Conclusion

Although rare, SDP should be accurately diagnosed and treated in the ED and should not be misdiagnosed as patellar tendon rupture. SDP commonly occurs in individuals with osteoarthritis; however, young patients with direct trauma to the knee may also present with this condition.
